# Evidence based practice in postgraduate healthcare education: A systematic review

**DOI:** 10.1186/1472-6963-7-119

**Published:** 2007-07-26

**Authors:** Gemma Flores-Mateo, Josep M Argimon

**Affiliations:** 1 Unitat d'Epidemiologia, Salut Pública i Serveis Sanitaris, IDIAPJordi Gol, Barcelona, Spain; 2 Divisio d'avaluació, Servei Catala de la Salut, Barcelona, Spain

## Abstract

**Background:**

Training in Evidence-Based Practice (EBP) has been widely implemented throughout medical school and residency curricula. The aim of this study is to systematically review studies that assessed the effectiveness of EBP teaching to improve knowledge, skills, attitudes and behavior of postgraduate healthcare workers, and to describe instruments available to evaluate EBP teaching.

**Methods:**

The design is a systematic review of randomized, non-randomized, and before-after studies. The data sources were MEDLINE, Cochrane Library, EMBASE, CINAHL and ERIC between 1966 and 2006. Main outcomes were knowledge, skills, attitudes and behavior towards EBP. Standardized effect sizes (E-S) were calculated. The E-S was categorized as small (E-S < 0.2), small to moderate (E-S between 0.2 and 0.5), moderate to large (E-S between 0.51 and 0.79), large (E-S > 0.79). Reliability and validity of instruments for evaluating education were assessed. Studies excluded were those that were not original, performed in medical students, focused on prescribing practices, specific health problems, theoretical reviews of different components of EBP, continuing medical education, and testing the effectiveness of implementing guidelines.

**Results:**

Twenty-four studies met our inclusion criteria. There were 15 outcomes within the 10 studies for which E-S could be calculated. The E-S ranged from 0.27 (95%CI: -0.05 to 0.59) to 1.32 (95%CI: 1.11 to 1.53). Studies assessing skills, behavior and/or attitudes had a "small to moderate" E-S. Only 1 of the 2 studies assessing knowledge had E-S of 0.57 (95 CI: 0.32 to 0.82) and 2 of the 4 studies that assessed total test score outcomes had "large" E-S. There were 22 instruments used, but only 10 had 2 or more types of validity or reliability evidence.

**Conclusion:**

Small improvements in knowledge, skills, attitudes or behavior are noted when measured alone. A large improvement in skills and knowledge in EBP is noted when measured together in a total test score. Very few studies used validated measures tests.

## Background

One of the most consistent findings in health-service research is the gap between best practice (as determined by scientific evidence) on the one hand and actual clinical care on the other [1,2]. Over the past decade, evidence-based clinical guidelines have become a major feature of healthcare provision. Biomedical researchers in many countries have established programs to garner evidence in the diagnosis and treatment of health problems, and to disseminate these guidelines in order to improve the quality of care provision. However, several studies have suggested that clinical use of these guidelines does not occur, that between 10 and 40% of patients do not receive care based on current scientific evidence, and that ≥ 20% of care provided is not needed or is potentially harmful to the patients [1,3-5].

A strategy to reduce these deficits in care provision is to increase the number of Evidence Based Practice (EBP) training programs [6-8]; their goal being to improve outcomes for patients by increasing postgraduate health care knowledge, skills and attitudes towards EBP [9]. However, published reports on effectiveness of these training schemes have shown conflicting results [10-13].

A crucial aspect in evaluating education programs is the choice of instrument for evaluating the effect of the educational training [14]. The rigor with which investigators and educators construct and/or administer the instrument could affect the reliability, validity and feasibility of the evaluation [14,15]. As such, a systematic and comprehensive review of existing instruments is necessary so as to describe the relationships between different educational instruments and the effectiveness of an EBP course in increasing knowledge, skills, attitudes and behavior in EBP and, as such, to be able to select the instrument that best assesses effectiveness of EBP training.

Hence, the purpose of this present study was to perform a systematic review of the studies that had assessed the effectiveness of teaching EBP whose objectives were to improve knowledge, critical appraisal skills, attitudes and behavior of postgraduate healthcare workers. We examined, as well, the measures used to evaluate the effectiveness of the intervention, together with their reliability and validity.

## Methods

### Search strategy and study selection

We searched: (1) MEDLINE, (2) Cocharane Library, (3) EMBASE, (4) the Cumulative Index of Nursing and Allied Health Literature (CINAHL^®^) and ERIC. We designed a search strategy for MEDLINE, accessed via PubMed, for studies investigating the effectiveness of EBP training in clinical practice by using free text and the Medical Subject Headings (MeSH) terms evidence based medicine, evidence based health care, evidence based practice, critical appraisal, knowledge, attitude, skills, behavior, clinical competence, teach, education intervention, courses, journal club, workshops, multifaceted intervention, residents, physicians, nurses, health care professionals, postgraduates. The literature search period covered January 1966 through December 2006, with no language restrictions. Also, we reviewed the reference lists of the relevant original papers and reviews.

We aimed to identify all the randomized, non-randomized and before-and-after comparison studies that assessed the effectiveness of teaching EBP designed to improve knowledge, skills, attitudes and behavior in postgraduate healthcare workers. Our exclusion criteria were studies that focused on (a) prescribing; (b) specific health problems; (c) theoretical reviews of different components of EBP (searching skills, formulating questions); (d) continuing medical education in general (not specifically in EBP); (e) undergraduates; (f) testing the effectiveness of implementing guidelines; (g) evaluating teaching methods using IT devices (PDA or computer-based reminder); (h) no original studies; and (i) medical students. When several papers were published from the same population, the publication with the longest follow-up was preferred.

### Data abstraction

Two investigators (G.F-M., J.M.A) independently abstracted the articles that met the selection criteria. Discrepancies were resolved by consensus. We reviewed each article that met the selection criteria and abstracted the data by using standardized data abstraction forms. Data abstracted were author, year of publication, country, design, participants (discipline and level), sample size, outcome, EBP intervention, duration and frequency of intervention, instruments for evaluating education, feasibility, and the types of reliability and validity assessed.

Feasibility was defined as documentation of some measure of ease of implementation of the questionnaire; time required to administer instrumentation, time required to score instrumentation, and the costs involved in administration and scoring. Reliability is concerned with that portion of measurement that is due to permanent effects which persist from sample to sample. Two broad types of reliability were abstracted: test-retest score or temporal stability and internal consistency. Types of validity assessed were those based on: content, internal structure (internal consistency and dimensionality), and relationships with other variables (responsive and discriminative criteria).

We assigned the types of outcome to the following categories: knowledge of EBP, skills defined as the participant applying knowledge by performing EBP steps in some scenarios, attitudes towards EBP, behavior defined as actual performance of EBP in practice. When two or more outcomes were combined in a score, we described this as a total test score.

We used the recommended questions for appraising reports of medical education interventions to assess study quality [14].

### Data synthesis

For those outcomes in which it was possible, we calculated an effect-size (E-S) for each outcome category, and are measures of the magnitude of an intervention effect [16]. The E-S is the difference in means divided by the square root of the pooled-group variances. Unless common metric units were used, this provides different units for outcomes measured. Converting the effect of the different studies to E-S enables comparisons to be made between studies. E-S calculations were made using the effect size generator software program [17]. The E-S was defined as "small" (E-S < 0.2), "small to moderate" (E-S between 0.2 and 0.5), "moderate to large" (E-S between 0.51 and 0.79), "large" (E-S > 0.79). We could not use meta-analysis of E-S for several reasons: (a) because the heterogeneity and diversity of outcomes reported do not allow for a clear metric scale to be used across the studies; (b) important information necessary for pooling studies (such as variance estimates) was missing in many studies; and (c) the diversity of studies (including populations, interventions and follow-up time) was not amenable to pooling.

We assessed publication bias by using the Begg and the Egger tests test and funnel plots, which graphically display the magnitude of the effect estimated as the inverse of variance of the study. All statistical analyses were conducted by using Stata software version 9.0 (STATA Corp, College Station, TX) and with S-PLUS version 7 (Insightful Corporation, Seattle, WA).

## Results

### Study characteristics

**Figure 1:**
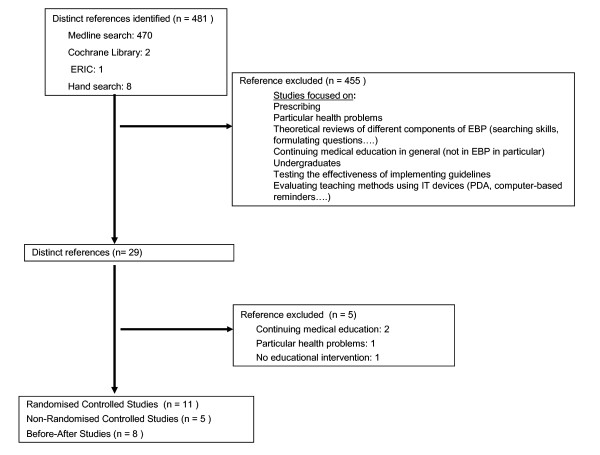
Study selection process.

We identified 481 published articles based on our search criteria. Following a review of the abstracts, we retrieved the full text of 29 and assessed them for information on effectiveness of EBP training in postgraduate healthcare workers. After applying the full review, 24 reports were finally included in the current evaluation (Figure 1).

The studies were published between 1988 and 2006 (see Additional file 1). There were 11 randomized controlled trials (RCT) [18-28], 5 non-randomized clinical trials (NRCT) [29-33] and 8 before-after studies [34-41]. Studies were geographically heterogeneous, and sample sizes varied considerably (between 12 and 800 subjects). In most of the studies the population was residents in medicine. Teaching methods included workshops, multifaceted intervention, internet-based intervention or journal club. The journal club was the most common format [18,21,30,31,35]. The duration of the teaching schedules ranged from 15 minutes to several years.

**Figure 2:**
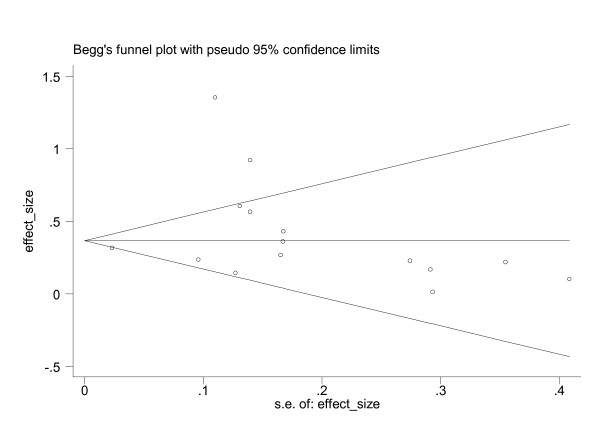
Funnel-plot of the effect size of size of randomized-controlled trials, non randomized-controlled trials and before-after studies.

Both the Begg and the Egger tests were significant (p < 0.05) and the funnel plot did not suggest any publication or related bias (Figure 2).

### Characteristics of EBP evaluation instruments

We found 22 instruments for evaluating education in EBP with two instruments being used in more than one study [33,36,38,40]. Feasibility of implementation was poorly reported for all instruments. None reported the time required for administering or scoring the instruments and none estimated the financial cost of implementation. Ten instruments (45.4%) were validated with at least two or more types of evidence of validity or reliability. The responsive validity was the one most commonly tested (90.9%) followed by discriminative (27.3%) and content validity (27.3%) (Table 1)

**Table 1:** Psychometric characteristics of educational instruments

Characteristics	Tested; n = 22 N (%)
Validity	
○ Content	6 (27.3)
○ Discriminative	6 (27.3)
○ Responsive	20 (90.9)
Reliability	
○ Cronbach Alpha	5 (22.7)
○ Kappa	2 (0.09)
○ Intra-class correlation	3 (13.6)
Instruments with ≥2 types of validity and reliability test	10 (45.4)

Categories are not mutually exclusive

### Assessment of Outcomes

There were 15 outcomes within the 10 studies for which E-S could be calculated [18,20,26,30,31,33,34,36-38] (Figure 2). Of these, 4 had a non-significant E-S [20,30,31,33]. The E-S ranged form 0.27 (95%CI: -0.0 to 0.59) for attitudes outcome [20] to 1.32 (95%CI: 1.11 to 1.53) for total test score [36] (Figure 2).

**Figure 3:**
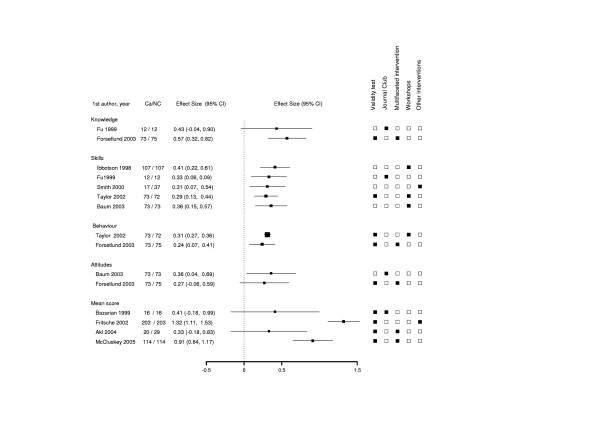
Forest Plot of the effect size (E-S) of randomized-controlled trials, non randomized-controlled trials and before-after studies. E-S corresponds to magnitude of an intervention effect. Boxes are the E-S estimates from each study. The horizontal bars are 95%CI. The size of the box is proportional to the weight of the study. The studies are sorted by weight in the plot. The table on the right side of the graph indicates whether two or more types of validity were used and what kind of intervention was used: Journal Club or workshops, or multifaceted intervention, or other interventions. ■ Yes; □ No.

Within the outcomes groups, 2 of the total test score outcomes had a significant E-S > 0.79. Five studies assessed skills, two assessed behavior and two assessed attitudes. All had a "small to moderate" E-S (range: 0.2 to 0.5). One of the two studies which assessed knowledge had E-S of 0.57 (95%CI: 0.32 to 0.82) [20] defined as "moderate to large", while the other study had a "small to moderate" E-S [30]. None of the knowledge, skills, attitudes and behavior outcomes had E-S > 0.79 (Figure 3).

We found that of the different types of intervention, the workshop was the most frequent intervention (35.3%), followed by multifaceted intervention (29.6%) (see Additional file 1 and Figure 2).

### Quality assessment

We used an adaptation of the quality measure from Reed et al. [14]. We examined 13 criteria of study quality (see Additional file 2). On average, the studies met more than half of the quality criteria. Only two studies met the criteria "Are long term effects assessed?"[38,42] and only one study did not meet the criteria "Is validity of instruments reported?

## Discussion

This review sought to identify those studies that examined the effectiveness of EBP education in improving knowledge, skills, behavior and attitudes in EBP in postgraduate health care. This is important from the medical education standpoint with intervention as a means of improving the quality of care provision. In our review we identified a small significant improvement in skills, knowledge, behavior and attitudes after EBP intervention. We identified a large improvement (E-S > 0.79) in EBP when measured as a total test score. Two of the four studies [36,38] included that had measured total test score had shown an E-S of up to 0.79. Both studies had used a validation test with high reliability and validity in assessing knowledge and skill in all the usual domains of evidence based medicine by asking focused questions, by searching for good answers, by critiquing literature, and by applying the conclusions to practice[36,43].

However, the poor quality of these studies precludes conclusions being made on the E-S of improving knowledge, skills, attitudes or behavior following EBP education. Of the 21 studies, 7 were before-after studies and did not employ a non-intervention group for comparison. The majority of the studies had small sample size; median of 59 participants (range, 12–800). Many studies provide little detail on how the questionnaires were developed and validated, how the questionnaires were administrated and how long before the intervention. All the studies were conducted in North America, the United Kingdom, Australia, Germany and Hong Kong, and do not accurately reflect developing countries. Only two studies were designed to assess long-term effect on skills[26,38] while the rest of the studies assessed short-term learning. The studies in this review were not able to distinguish whether the observed outcomes were the result of receiving the intervention or the desire of the health care professional to change. Integrating theories of behavior change into education programs is one of the keys for successful education development. Sustained learner behavior and change in attitude of individuals with high motivation to learn were more active in the education programs[44].

Our results are consistent with a previous systematic study [11] which found small changes in knowledge at the level of the resident but, in contrast, this improvement was high in undergraduate medical students. And another systematic review [10] showed that standalone teaching improved knowledge but not skills, attitudes or behavior. Finally, a systematic review of the effectiveness of critical appraisal skills in the training of clinicians showed an overall improvement of 68% in assessed outcomes following intervention, but only one study used a randomized controlled design and the methodological quality of the studies included was poor [13].

This review focused as well on examining which studies had used a validation instrument to assess the effectiveness of the intervention. Changes in health cares' knowledge and skills are relatively easy to detect with validation instruments, but changes in behavior and attitudes are more difficult to measure. Several authors have proposed assessment in the practice setting, or by conducting qualitative studies [37,45]. None of the studies reported health care outcomes and none of had documented any measure of ease-of-implementation, time required to administer the instrument, time required to score the instrument or the costs of administering and of scoring. Only 9 of the 19 instruments (47.4%) revised 2 or more types of validity or reliability. Choice of measurement method is a crucial step in the evaluation of educational interventions because many evaluation methods are not sensitive enough to measure the effectiveness of the interventions, and which could lead to incorrect interpretation of results [14]. Also, the use of validated tests enable comparison of results to be made between different studies[14,46]. This is an important area for further research, and one in which healthcare research workers need to document the reliability and validity of existing measures, rather than to continue developing new instruments for each new project.

As with our present review, but with a smaller number of studies reviewed, only one other systematic review of EBP teaching had addressed the effectiveness of educational interventions and had included detailed analysis of the evaluation instrument [12]. Another systematic review assessed the available EBP teaching instrument methods but did not report on the effectiveness of EBP teaching [15]. The results of our systematic review confirm the findings of previous assessments indicating that few types of validity and of reliability evidence are contained in the instruments evaluating education in EBP.

There are several limitations in this current review. The eligibility of the studies in our systematic evaluation was limited to published reports. Our resources did not permit an extensive search of the literature outside of the stated databases. However, a study has shown that results of reviews incorporating non-catalogued literature do not differ substantially from those reviews that do contain them [47], and no significant publication bias was found in our analyses. One of the strengths of the present systematic review is the use of the effect-size; the goal being to obtain a standardized outcome measure which would enable comparisons to be made of the results from different studies.

The results of this review provide an outline of common themes for future research: (a) randomized controlled studies with appropriate study sample size and using validated tests are warranted in assessing the effectiveness of EBP training; (b) developing and trans-culturally adapted instruments with strong evidence of validity and reliability and whose evaluation domains correspond to assessing knowledge, skills, attitudes and behavior in EPB; (c) studies to examine the importance of personality traits and intention-to-change of health-care professionals; (d) studies to improve outcomes for patients by increasing physicians' knowledge, skills and attitudes towards EBP; (e) integration of theories of behavior-change into education programs and to measure the effect on clinical competence.

## Conclusion

Randomized controlled trials, non-randomized controlled trials and before-after studies showed a small improvement in knowledge, skills, attitudes and behavior following EBP, together with a large improvement in knowledge and skills when measured as a total test score. However, the quality of the evidence precludes practical recommendations to be introduced in EBP education in postgraduate health-care professionals. More research into education in medicine is needed. Greater collaboration with organizations and individuals interested in preserving standards in academic medicine is required. Programs of training health-care professionals have responsibility for education and research. These programs must stimulate interest in EBP education and must evaluate these interventions. EBP education and other types of medical education interventions should be evaluated in a similar manner as that expected for interventions such as drug therapy or diagnostic studies.

## Competing interests

The author(s) declare that they have no competing interests.

## Authors' contributions

JMA and GFM were equally responsible for design, quality assessment, review of studies, and preparation of the manuscript.

## Pre-publication history

The pre-publication history for this paper can be accessed here:



## Supplementary Material

Additional file 1 Characteristics of studies assessing effectiveness of teaching critical appraisal skills. this table provided information about the assessing effectiveness of teaching critical appraisal skills. Click here for file

Additional file 2 Quality criteria for evaluating studies. This table ass the quality criteria of studies included in the systematic review. Click here for file
