# Supplementary material for: Evidence based practice in postgraduate healthcare education: A systematic review
Source: BMC Health Serv Res. 2007 Jul 26;7:119. doi: 10.1186/1472-6963-7-119 (PMC1995214; doi:10.1186/1472-6963-7-119)
Supplement: Additional file 1 — Characteristics of studies assessing effectiveness of teaching critical appraisal skills. this table provided information about the assessing effectiveness of teaching critical appraisal skills. [file 1472-6963-7-119-S1.doc]

**Table 1. Characteristics of studies assessing effectiveness of teaching critical appraisal skills**

| Author,  Year [ref] | Country | Design | Population | Sample | Intervention | Duration and frequency of intervention | Outcomes | Description of instrument for evaluating | Reliability | Validity |
| --- | --- | --- | --- | --- | --- | --- | --- | --- | --- | --- |
| Linzer,  1988 [21] |  | RCT | Medical Interns | 44 | Journal Club, Free text critical appraisal | 1h / week for 1 year | Knowledge  Skills | MCQ (15) | Yes (not reported) | Responsive |
| Haynes, 1993[28] | Canada | RCT | Physicians from clinical departments | 392 | Course and clinical preceptor | Two hour course | Skills | Search output scored | Kappa 0.79 | Content  Discriminative  Responsive |
| Green,  1997 [29] | USA | CT | Primary Care Residents | 34 | Multifaceted intervention | 1 hour / week for 7 weeks | Behavior  Skills | MCQ (9) and free text responses | ICC 0.87 | Content  Responsive |
| Ibbotson, 1998 [34] | UK | Before-after | Medical research, managerial and nursing from Scotland | 164 | Workshops | Two hour sessions | Knowledge Skills | Questionnaire and Semi-structured interview | N.R | Responsive |
| Stevermer,  1999 [24] | USA | RCT | Family Medicine Residents | 59 | Educational presentation | 15 minutes | Skills  Knowledge | Percentage of journal article aware | N.R | Responsive |
| Fu,  1999 [30] | UK | CT | Psychiatry Residents | 24 | Journal club | 1.5 hour / week for 12 weeks | Skills  Knowledge | MCQ (19), survey and clinical case | NR | Responsive |
| Bazarian, 1999 [31] | USA | CT | Emergency Medicine Residents | 32 | Journal Club | 1h /month for 1 year | Knowledge Skills | Free text responses | ICC (not reported) | Responsive |
| Smith,  2000 [26] | USA | RCT | First-year internal medicine residents | 55 | Course and Computer lab | 2h/ week and 1.5 computer lab for 7 weeks | Knowledge Skills  Behavior | Clinical case test (5)  Self-reported EBM skills | N.R | Responsive |
| Kellum, 2000 [35] | USA | Before-after | Fellows in critical care medicine | 12 | Journal Club | 1h/ 2 sessions | Knowledge Skills | Test and self-reported | N.R | Responsive |
| Villanueva, 2001[27] | Australia | RCT | Health Care Professionals | 52 | Revised form | NR | Skills  Behavior | Search output scored | N.R | Responsive |
| Cabell,  2001 [25] | UK | RCT | Medical residents | 48 | Multifaceted intervention | NR | Skills | Collected data from the OVID system | N.R | Responsive |
| Fritsche, 2002 [36] | Germany | Before-after | Experts in evidence based medicine and third year medical students | 266 | Course | Three days | Skills Knowledge | Berlin test. MCQ (30) | Alpha 0.75 and 0.82 | Responsive  Discriminative |
| Cheng,  2003 [19] | Hong Kong | RCT | Hospital Clinicians, health professionals | 800 | Workshops | 3 hours | Knowledge  Skills  Attitudes  Behavior | MCQ or true-false questions | NR | NR |
| Forsetlund, 2003 [20] | Norway | RCT | Public Health Physicians | 148 | Multifaceted intervention | NR | Knowledge  Attitudes | MCQ | Alpha 0.83 to 0.87 | Responsive  Discriminative |
| Ross,  2003 [32] | USA | CT | Primary Care Residents | 48 | Workshops | 1h/ week for 10 weeks | Knowledge | MCQ (50) analysis of recorded | N.R | Content  Responsive |
| Baum,  2003 [37] | USA | Before-after | Internal Medicine and Pediatric residents | 73 | Workshop | Half-day | Attitudes | MCQ (9) and self-reported | NR | Responsive |
| Taylor,  2004 [18,47] | UK | RCT | General Practitioners; Hospital physicians; Professionals allied to medicine Healthcare managers | 145 | Workshops | 1 hour / week for 10 weeks | Knowledge  Attitude  Skills | MCQ ( 31 ) | ICC 0.70 to 0.86 | Content  Discriminative  Responsive |
| Macrae, 2004 [22] | Canada | RCT | General surgeons | 99 | Internet-based intervention | 6 months | Skills | Questionnaire | Alpha 0.77  Inter-rater 0.93 | Content  Discriminative |
| Akl,  2004 [33] | USA | CT | Residents and Medical students | 40 | Multifaceted intervention | Two-week rotation | Skills  Knowledge | Berlin test | Alpha 0.75 | Content  Responsive |
| Lucas 2004[41] | USA | Before-after | 12 hospital’s departments medicine | 33 | Literature search course | 24 hours/ one day | Behavior | Interviews | NR | Responsive |
| McCluskey, 2005 [38] | Australia | Before-after | Occupational therapists | 114 | Multifaceted intervention | Two days | Skills  Knowledge  Attitudes  Behavior | Fresno test, and Firedman test | Alpha 0.72 to 0.84 | Responsive  Content  Discriminative |
| Straus,  2005 [39] | UK | Before-after | Physicians and Medicine residents | 47 | Multifaceted intervention | 1h for 7 session | Therapy supported by evidence | Evaluate summaries for patients admitted | NR | Responsive |
| Schilling, 2006 [23] | USA | RCT | Family Medicine | 207 | Web-based curriculum | 6-week | Knowledge  Skills | Survey and Search strategies measured | NR | Responsive  Inter-rater 0.66 to 0.81 |
| Dinkevich, 2006 [40] | USA | Before-after | Pediatric residents | 69 | Seminars | 2h/ week for 4 weeks | Knowledge Skills | Fresno test | Kappa 0.74 | Responsive  Discriminative |

Notes to Table 1

NR: Not reported. ICC: Intra-class correlation. MCQ: multiple choice questions (number of questions in brackets): RCT: randomized controlled trial. CT: controlled trial
