# Supplementary material for: Evidence based practice in postgraduate healthcare education: A systematic review
Source: BMC Health Serv Res. 2007 Jul 26;7:119. doi: 10.1186/1472-6963-7-119 (PMC1995214; doi:10.1186/1472-6963-7-119)
Supplement: Additional file 2 — Quality criteria for evaluating studies. This table ass the quality criteria of studies included in the systematic review. [file 1472-6963-7-119-S2.doc]

**Additional file 2. Quality criteria for evaluating studies**

| Study reference number: | [21] | [28] | [29] | [34] | [24] | [30] | [31] | [26] | [35] | [27] | [25] | [36] | [19] | [20] | [32] | [37] | [18] | [22] | [33] | [41] | [38] | [39] | [23] | [40] |
| --- | --- | --- | --- | --- | --- | --- | --- | --- | --- | --- | --- | --- | --- | --- | --- | --- | --- | --- | --- | --- | --- | --- | --- | --- |
| Is the study purpose easily identified? | ■ | ■ | ■ | ■ | ■ | ■ | ■ | ■ | ■ | ■ | ■ | ■ | ■ | ■ | ■ | ■ | ■ | ■ | ■ | ■ | ■ | ■ | ■ | ■ |
| Are objectives congruent with intervention and evaluation? | ■ | ■ | ■ | ■ | ■ | ■ | ■ | ■ | ■ | ■ | ■ | ■ | ■ | ■ | ■ | ■ | ■ | ■ | ■ | ■ | ■ | ■ | ■ | ■ |
| Is study design appropriate for question? | ■ | ■ | ■ | ■ | ■ | ■ | ■ | ■ | ■ | ■ | ■ | ■ | ■ | ■ | ■ | ■ | ■ | ■ | ■ | ■ | ■ | ■ | ■ | ■ |
| Is there a similar comparison group? | ■ | ■ | □ | □ | ■ | □ | □ | ■ | □ | ■ | ■ | □ | ■ | ■ | □ | □ | ■ | ■ | □ | □ | □ | □ | ■ | □ |
| Are raters blinded with respect to group assignment? | ■ | ■ | ■ | □ | ■ | □ | □ | ■ | □ | □ | □ | □ | ■ | □ | □ | ■ | ■ | □ | □ | □ | □ | ■ | □ | ■ |
| Is study design described in sufficient detail to be replicated? | ■ | ■ | ■ | ■ | ■ | ■ | ■ | ■ | ■ | ■ | ■ | ■ | ■ | ■ | ■ | ■ | ■ | ■ | ■ | ■ | ■ | ■ | ■ | ■ |
| Are long term effects assessed? | □ | □ | □ | □ | □ | □ | □ | ■ | □ | □ | □ | □ | □ | □ | □ | □ | □ | □ | □ | □ | ■ | □ | □ | □ |
| Are confounding variables controlled-for by design or analyses? | ■ | ■ | □ | □ | ■ | □ | □ | ■ | ■ | ■ | ■ | □ | ■ | ■ | ■ | □ | ■ | ■ | □ | □ | □ | □ | ■ | □ |
| Has power analysis been conducted to determine sample size? | ■ | ■ | □ | □ | □ | ■ | □ | □ | □ | □ | □ | □ | ■ | ■ | □ | □ | ■ | ■ | □ | ■ | □ | □ | □ | □ |
| Are teaching methods described in enough detail to replicate? | ■ | ■ | ■ | ■ | ■ | ■ | ■ | ■ | ■ | ■ | ■ | ■ | ■ | ■ | ■ | ■ | ■ | ■ | □ | □ | ■ | ■ | ■ | ■ |
| Is reliability of instruments reported? | ■ | ■ | ■ | □ | □ | □ | ■ | □ | □ | □ | □ | ■ | □ | ■ | □ | □ | ■ | ■ | ■ | □ | ■ | □ | □ | ■ |
| Is validity of instruments reported? | ■ | ■ | ■ | ■ | ■ | ■ | ■ | ■ | ■ | ■ | ■ | ■ | □ | ■ | ■ | ■ | ■ | ■ | ■ | ■ | ■ | ■ | ■ | ■ |
| Are statistical tests described? | ■ | ■ | ■ | ■ | ■ | ■ | ■ | ■ | ■ | ■ | ■ | ■ | ■ | ■ | ■ | ■ | ■ | ■ | ■ | ■ | ■ | ■ | ■ | ■ |

■ Yes; □ No
